# A Novel Fluorescent Probe for the Detection of Hydrogen Peroxide

**DOI:** 10.3390/bios13060658

**Published:** 2023-06-16

**Authors:** Kangkang Wang, Tingting Yao, Jiayu Xue, Yanqiu Guo, Xiaowei Xu

**Affiliations:** 1 State Key Laboratory of Natural Medicines, Key Lab of Drug Metabolism and Pharmacokinetics, China Pharmaceutical University, Nanjing 210009, China; 1822010275@stu.cpu.edu.cn (K.W.); 3221071574@stu.cpu.edu.cn (T.Y.); 3322071703@stu.cpu.edu.cn (J.X.); 2 Nanjing Luhe People’s Hospital, Nanjing 210009, China; guoyanqiuqiu@outlook.com

**Keywords:** fluorescent probe, hydrogen peroxide, boronic acid

## Abstract

Hydrogen peroxide (H_2_O_2_) is one of the important reactive oxygen species (ROS), which is closely related to many pathological and physiological processes in living organisms. Excessive H_2_O_2_ can lead to cancer, diabetes, cardiovascular diseases, and other diseases, so it is necessary to detect H_2_O_2_ in living cells. Since this work designed a novel fluorescent probe to detect the concentration of H_2_O_2_, the H_2_O_2_ reaction group arylboric acid was attached to the fluorescein 3-Acetyl-7-hydroxycoumarin as a specific recognition group for the selective detection of hydrogen peroxide. The experimental results show that the probe can effectively detect H_2_O_2_ with high selectivity and measure cellular ROS levels. Therefore, this novel fluorescent probe provides a potential monitoring tool for a variety of diseases caused by H_2_O_2_ excess.

## 1. Introduction

Reactive oxygen species (ROS) are chemically reactive substances that contain oxygen. It contains superoxide anion (^•^O_2_^−^), hydrogen peroxide (H_2_O_2_), hydroxyl radical (^•^OH), ozone (O_3_), and singlet oxygen (^1^O_2_). Because they contain unpaired electrons and have high chemical reactivity, they play an important role in a variety of physiological and pathological processes [1,2,3,4,5,6,7]. Among them, H_2_O_2_, which is continuously produced by basic cellular processes such as protein folding, is a kind of enzyme-catalyzed active oxygen metabolism by-product [5,8] and can serve as a key modulator in many oxidative stress-related statuses [9]. The excessive production and accumulation of hydrogen peroxide in the body can lead to various diseases such as cancer, aging, asthma, and cardiovascular and neurodegenerative diseases [10,11]. Up until now, the important role of H_2_O_2_ in human health and various diseases has not been fully revealed, so it is of great significance to develop a sensitive and effective method to detect the level of H_2_O_2_. 

Currently, the main detection methods for H_2_O_2_ include the fluorescence probe method, spectrophotometry, electrochemical method, colorimetric method, etc. [12,13,14,15,16,17,18,19,20,21,22,23,24]. Sample preparation for research methods such as spectrophotometry, electrochemistry, and colorimetry is complex and cannot dynamically reflect changes in H_2_O_2_ levels or effectively detect the concentration of H_2_O_2_ in living cells. In contrast, fluorescence probe methods provide a powerful method for monitoring H_2_O_2_ levels in the living system [25,26]. Fluorescent probes are usually composed of fluorescent groups, detection groups, and connecting groups. By connecting different fluorescent groups and different detection groups, it is possible to design fluorescent probes with diverse performances to meet various detection requirements. Therefore, using fluorescence probes to detect hydrogen peroxide related to many diseases in the human body is still an essential technology. In 2003, the first boric acid-based H_2_O_2_ fluorescence probe was reported [27]. Studies have revealed that the probe is effective at detecting H_2_O_2_. Boric acid or borate esters are frequently utilized as hydrogen peroxide reaction components because a significant number of studies have demonstrated that probes based on the oxidation reaction of borate esters have superior selectivity for H_2_O_2_ than other ROS. The design of probe recognition groups has been verified for most classical fluorophores, such as coumarin [28], naphthalimide [29], and AIE mechanism fluorophores [30]. 

In order to meet the needs of identifying and characterizing the different sources and functions of hydrogen peroxide as a transient redox messenger, we designed and synthesized a novel fluorescent probe, YXSH, that combines the H_2_O_2_ reaction group arylboronic acid with fluorescein 3-Acetyl-7-hydroxycoumarin as a specific recognition group for the selective detection of hydrogen peroxide. The experimental results demonstrate that the probe can effectively detect H_2_O_2_ with high selectivity. Therefore, this novel fluorescent probe provides a potential monitoring tool for a variety of diseases caused by H_2_O_2_ excess.

## 2. Materials and Methods

### 2.1. Instruments and Reagents

All chemical reagents required for this study were purchased from Bidepharm Technology Co., Ltd (Bidepharm, Shanghai, China). No further purification was required. For the NMR spectra, ^1^H (300 MHz) and ^13^C (75 MHz) of the probe YXSH were collected using an AVANCE 300 MHz spectrometer. Fluorescence and UV-visible absorption spectra are measured by the Perkin Elmer Fluorescence Spectrometer FL6500 fluorescence spectrophotometer. High-resolution mass spectrometry (HRMS) data for the synthesis of the new compounds were determined using an Agilent 6500. High-performance liquid chromatography (HPLC) data were determined using the Agilent 1220 Infinity II. Fluorescent emission spectra were collected on a Perkin Elmer LS 55.

### 2.2. Synthesis of Compound ***2***

2,4-Dihydroxybenzaldehyde (279 mg, 2 mmol) and Ethyl acetoacetate (253 μL, 2 mmol) were dissolved in ethanol (6 mL), followed by a few drops of piperidine as a catalyst, and the reaction mixture was returned to 78 °C for 2 h to cool. Pour cold, dilute hydrochloric acid, filter the precipitate, rinse the precipitate with water, and recrystallize the purified residue from methanol to obtain the product; the product is a light yellow crystal (286 mg, 70%). The ^1^H NMR (300 MHz, DMSO-*d*_6_) *δ*(ppm): 11.15 (s, 1H), 8.60 (s, 1H), 7.78–7.81 (d, *J* = 8.4 Hz, 1H), 6.84–6.87 (dd, *J*_1_ = 8.7 Hz, *J*_2_ = 2.4 Hz, 1H), 6.75–6.76 (d, *J* = 2.1 Hz, 1H), and 2.55 (s, 3H). HRMS C_11_H_8_O_4_, *m*/*z*: [M+H]^+^ calcd 205.05, found 205.05.

### 2.3. Synthesis of Probe YXSH

3-Acetyl-7-hydroxy-2H-chromen-2-one (202 mg, 1 mmol), 4-(Bromomethyl)phenylboronic acid (219 mg, 1 mmol), Anhydrous K_2_CO_3_(963 mg, 7 mmol), and acetone (15 mL) were added to the flask, the reaction mixture was reflow at 55 °C for 14 h, the reaction mixture was cooled and filtered, the solvent was removed by spin evaporation, DCM extraction was carried out, the organic phase was cleaned in saturated salt water, dried on anhydrous sodium sulfate, and then filtered, and the volatiles were removed under vacuum. The residue was purified by silica gel column chromatography to obtain a crude product, which was then recrystallized with DCM n-hexane to produce a bright yellow powder (179 mg, 53%). The ^1^H NMR (300 MHz, DMSO-*d*_6_) *δ*(ppm): 8.64 (s, 1H), 8.11 (s, 2H), 7.80–7.96 (m, 3H), 7.43–7.45 (d, *J* = 7.5 Hz, 2H), 7.11 (t, *J* = 10.8 Hz, 2H), 5.28 (s, 2H), and 2.56 (s, 3H). The ^13^C NMR (75 MHz, DMSO-*d*_6_) *δ*(ppm): 195.28, 164.30, 159.39, 157.50, 148.12, 138.15, 134.82, 132.76, 127.40, 120.95, 114.57, 112.47, 101.62, 70.68, 49.11, and 30.63. HRMS C_18_H_15_BO_6_, *m*/*z*: [M]^+^ calcd 338.10, found 338.34.

### 2.4. Stability Experiment with YXSH

Phosphate Buffered Saline (PBS) containing 100 μM H_2_O_2_ (from a 100 mM stock solution in H_2_O) and 10 μM YXSH (1 mM stock solution in DMSO) was incubated for 30 min at 37 °C on a shaker in the dark. H_2_O_2_ was first added to PBS, and then YXSH was added. The reaction solution was added to a 96-well plate (each well containing 200 μL), and six replicate wells were set up. Assayed it every 20 min for 10 h.

### 2.5. Sensitivity Experiment with YXSH

PBS separately containing 0, 1, 2, 5, 8, 10, 20, 30, 40, 50, 80, and 100 μM H_2_O_2_ (from a 100 mM stock solution in H_2_O) and 1 μM YXSH (1 mM stock solution in DMSO) was incubated for 30 min at 37 °C on a shaker in the dark. H_2_O_2_ was first added to PBS, and then YXSH was added. The reaction solution was added to a 96-well plate (each well containing 200 μL), and three replicate wells were set up. Assayed it immediately.

### 2.6. Selectivity Experiment of YXSH

PBS separately containing 100 μM cations (Na^+^, K^+^, Fe^2+^, Mg^2+^, and Cu^2+^), anions (HCO_3_^−^, Cl^−^, OH^−^, and SO_4_^2−^), amino acids (Arg, Cys, Ala, and Gly), L-GSH, C_10_H_6_O_4_, TBAF (from a 100 mM stock solution in H_2_O/DMSO), and H_2_O_2_ with 10 μM YXSH (1 mM stock solution in DMSO) was incubated for 30 min at 37 °C on a shaker in the dark. YXSH was added to PBS at the end. The reaction solution was added to a 96-well plate (each well containing 200 μL), and three replicate wells were set up. Assayed it immediately. 

### 2.7. Cell Culture 

Human NSLCS A549 cell lines were obtained from the Shanghai Cell Bank of the Chinese Academy of Sciences (Shanghai, China). The A549 cell line was cultured in Dulbecco’s modified Eagle’s medium (DMEM, Gibco, Grand Island, NY, USA) supplemented with 10% fetal bovine serum (FBS, Prime, FSP500, ExCell Bio, Shanghai, China) and 1% penicillin/streptomycin (Gibco, Grand Island, NY, USA). A549 cell lines were grown at 37 °C in a 5% CO_2_ and 95% air-humidified atmosphere and sub-cultured every 2−3 days.

### 2.8. Measurement of Intracellular ROS Levels 

A549 cells (5 × 10^4^ cells/glass) were seeded in a 4-Chamber Glass Bottom Dish. After 24 h, cells were incubated with lipopolysaccharide (LPS, *coli*. 0111:B4, Sigma, Shanghai, China) (2 μg/mL) for 2 h [31,32,33], followed by treatment with YXSH for 2 h. Finally, fluorescent images of cells were acquired on an LSM-700 Microscope (Zeiss, Jena, TH, Germany) with an objective lens (×40) using a green filter (excitation wavelength: 405 nm).

## 3. Results and Discussions

**Scheme 1:**
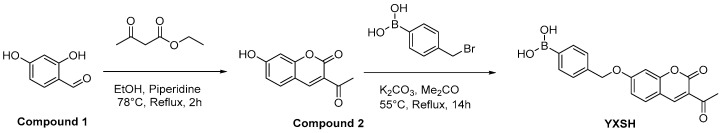
Synthetic route of the hydrogen peroxide probe.

Design and synthesis of compound YXSH. As shown in Scheme 1, our developed probe only involves two steps. Firstly, we synthesized 3-Acetyl-7-hydroxy-2H-chromen-2-one (compound **2**) using 2,4-Dihydroxybenzaldehyde and Ethyl acetate as raw materials. Then, we reacted arylboronic acid with compound **2** to obtain a probe, 3-Acetyl-7-[(4-boronyl)method]-2H-1-benzopyran-2-one (compound YXSH), that binds to H_2_O_2_, which is used for selective detection of hydrogen peroxide in living cells. The synthesis of compound YXSH has not been reported before, and the characteristic data of the obtained product can be found in the supplementary information.

**Figure 1:**
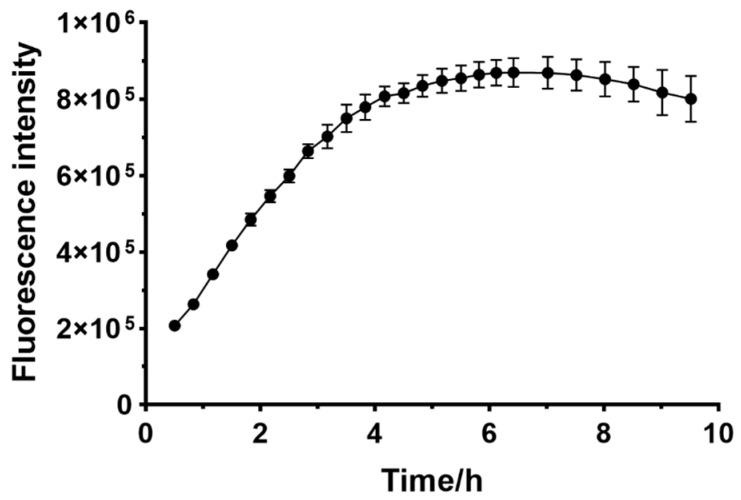
The fluorescence intensity changes versus the time of probe YSXH (10 μM) toward H_2_O_2_ (100 μM). λex = 415 nm and λem = 455 nm.

Stability and sensitivity measurements of YXSH. Firstly, the fluorescent spectra of the probe were recorded, and they showed a maximum fluorescent emission at 455 nm under excitation at 415 nm. Subsequently, the reaction time of the probe and the stability of the fluorescence were tested after the reaction. As shown in Figure 1, the fluorescence increased to 25% of the maximum intensity within 30 min and continued to increase, stabilizing after 5 h. This proved that YXSH has good stability, and the fluorescence was not easily quenched after the reaction with H_2_O_2_.

**Figure 2:**
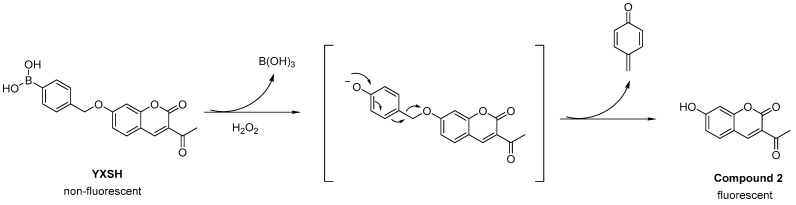
Proposed reaction mechanism of YXSH with H_2_O_2_.

Subsequently, we investigated the response mechanism of this probe and found that YXSH contains a boric acid group as both a reaction site and an electron-withdrawing group. The 3-Acetyl group is also an electron-withdrawing group, and the two electron-withdrawing groups weaken the fluorescence of the compound. However, when it reacts with hydrogen peroxide, the electron-deficient boric acid group becomes the electron-donating hydroxyl group, and the 3-Acetyl group acts as the electron-sucking group, forming a push–pull system. The intramolecular charge transfer (ICT) process was enhanced, and the reaction product compound **2** had stronger fluorescence emission than the probe (Figure 2). In order to verify the response mechanism of YXSH, liquid chromatography was used. High-performance liquid chromatography (Figure S4) showed that YXSH showed a signal peak at 28.768 min and compound **2** showed a signal peak at 7.982 min. After the reaction of YXSH with H_2_O_2_ for 30 min and 12 h, although the chromatographic baseline was not smooth, the YXSH signal peak decreased significantly, and new signal peaks appeared at 8.596 min (reaction time: 30 min) and 7.079 min (reaction time: 12 h). The retention time was almost consistent with compound **2**. Therefore, the experiment supports the fact that the structural transformation caused by the reaction of YXSH with H_2_O_2_ triggers the enhancement of the fluorescence signal, which proves our inference of the response mechanism of YXSH to H_2_O_2_ proposed in Figure 2.

**Figure 3:**
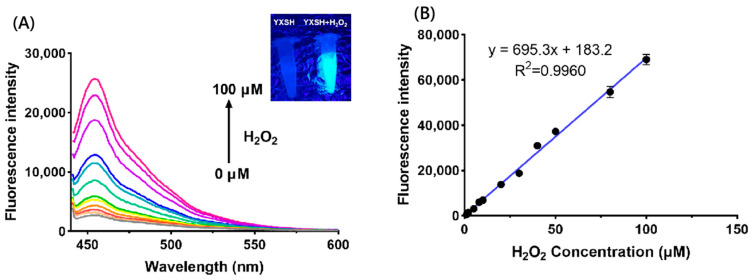
Quantitative measurements of YXSH fluorescent enhancement induced by different concentrations of H_2_O_2_. (**A**) Fluorescence spectra of probe YSXH in the presence of H_2_O_2_ (0, 1, 2, 5, 8, 10, 20, 30, 40, 50, 80, and 100 μM). (**B**) Linear regression plot of the fluorescent intensity of probe YSXH following incubation with increasing concentrations of H_2_O_2_ (1, 2, 5, 8, 10, 20, 30, 40, 50, 80, and 100 μM). The concentration of probe YSXH is 1 μM. λex = 415 nm and λem = 455 nm.

As shown in Figure 3A, the probe concentration was 1 μM, and the tested H_2_O_2_ concentrations ranged from 1 μM to 100 μM. As the concentration of H_2_O_2_ increases, the fluorescence intensity increases. As shown in Figure 3B, the titration curve of fluorescence intensity was plotted, which showed a good linear relationship, and the LOD was as low as 0.9 μM. This LOD was not that good compared with the previously reported probes, but it was low enough to detect H_2_O_2_ in cells.

**Figure 4:**
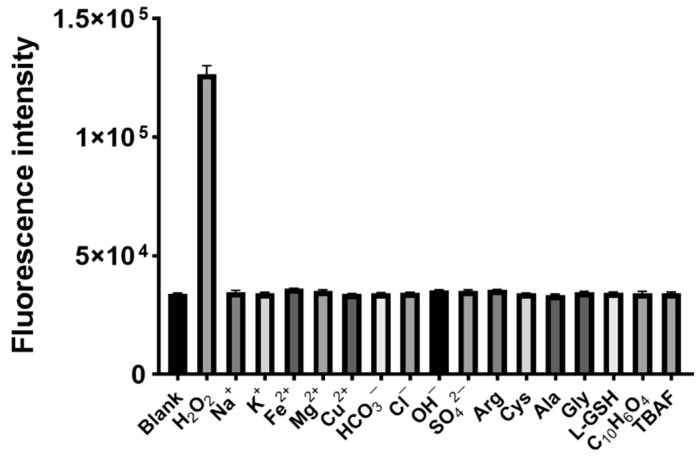
The fluorescence intensity changes of the probe YSXH (10 μM) in PBS buffer with the addition of different anions, cations, and amino acids. The concentration of each compound is 100 μM in PBS buffer (pH = 7.4).

Selectivity measurement of YXSH. As shown in Figure 4, cations (Na^+^, K^+^, Fe^2+^, Mg^2+^, and Cu^2+^), anions (HCO_3_^−^, Cl^−^, OH^−^, and SO_4_^2−^), amino acids (Arg, Cys, Ala, and Gly), L-GSH, C_10_H_6_O_4_, TBAF, and H_2_O_2_ were incubated with YSXH, respectively, and a blank control group was set up. The results showed that only the reaction of H_2_O_2_ with YSXH produced significant fluorescence under the PBS buffer, which proved the good specificity of YSXH. The above experimental data indicate that probe YXSH can react with H_2_O_2_ to generate strong fluorescent compounds with good selectivity, which can effectively characterize H_2_O_2_ and provide a potential monitoring tool for various diseases caused by excessive H_2_O_2_.

**Figure 5:**
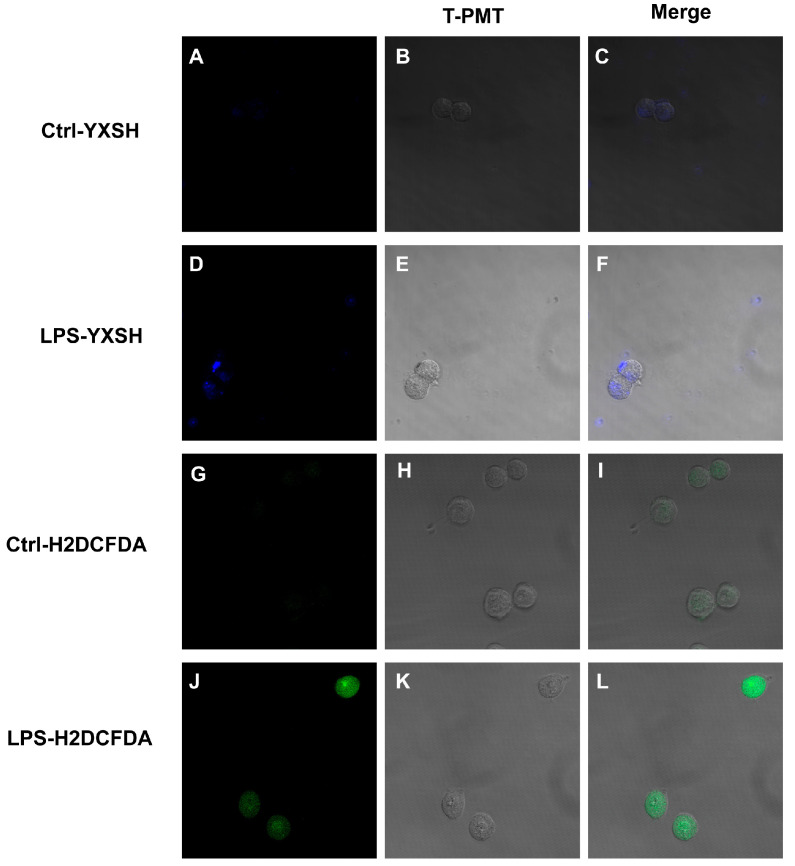
Fluorescence images of cellular ROS stimulated by LPS in A549 cells. A549 cells were incubated with 10 μM YXSH (**A**–**F**) or H2DCFDA (**G**–**L**) in the absence (**A**–**C**,**G**–**I**) or presence (**D**–**F**,**J**–**L**) of LPS (2 μg/mL). Bright-field images of cells were shown in (**B**,**E**,**H**,**K**). Merged fluorescent images of cells were shown in (**C**,**F**,**I**,**L**). Scale bar: 20 μm. λex = 405 nm (YXSH) or 488 nm (H2DCFDA).

Measurement of cellular ROS levels in A549 cells. YXSH (10 μM) was used to detect endogenous ROS in living A549 cells by stimulating cells with LPS at 2 μg/mL for 2 h. It shows that the fluorescence intensity in cells pretreated with LPS increased (Figure 5A–F). Meanwhile, LPS incubation can cause endogenous ROS in A549 cells to be elevated, as confirmed by the commercially available probe H2DCFDA (Figure 5G–L). After being treated with H2DCFDA (10 μM), the fluorescence intensity in cells pretreated with LPS is stronger than in the control group. The results showed that the YXSH succeeded in labeling endogenous ROS in A549 cells.

## 4. Conclusions

In summary, we have prepared a novel fluorescent probe, YXSH, that characterizes hydrogen peroxide. The probe combines the H_2_O_2_ reaction group arylboronic acid with fluorescein 3-Acetyl-7-hydroxycoumarin to form a specific recognition group for selective detection of hydrogen peroxide. Our relevant research data indicates that this probe can effectively characterize H_2_O_2_ with high selectivity and measure cellular ROS levels. As a result, this novel probe provides a potential monitoring tool for various diseases caused by excessive H_2_O_2_.

## Data Availability

Not applicable.

## Supplementary Materials

The following supporting information can be downloaded at: https://www.mdpi.com/article/10.3390/bios13060658/s1, Figure S1: ^1^H NMR spectrum of compound **2**. Figure S2: ^1^H NMR spectrum of YXSH. Figure S3: ^13^C NMR spectrum of YXSH. Figure S4: Liquid chromatography of YXSH, YXSH treated with H_2_O_2_, and compound **2**.
