# Supplementary material for: A Novel Fluorescent Probe for the Detection of Hydrogen Peroxide
Source: Biosensors (Basel). 2023 Jun 16;13(6):658. doi: 10.3390/bios13060658 (PMC10296328; doi:10.3390/bios13060658)
Supplement: Supplementary file 1 [file biosensors-13-00658-s001.zip › biosensors-2430189-supplementary.pdf]

biosensors

MDPI

Supporting Information

A Novel Fluorescent Probe for the Detection of Hydrogen Peroxide

Kangkang Wang [1,†], Tingting Yao [1,†], Jiayu Xue [1,†], Yanqiu Guo [2] and Xiaowei Xu [1,*]

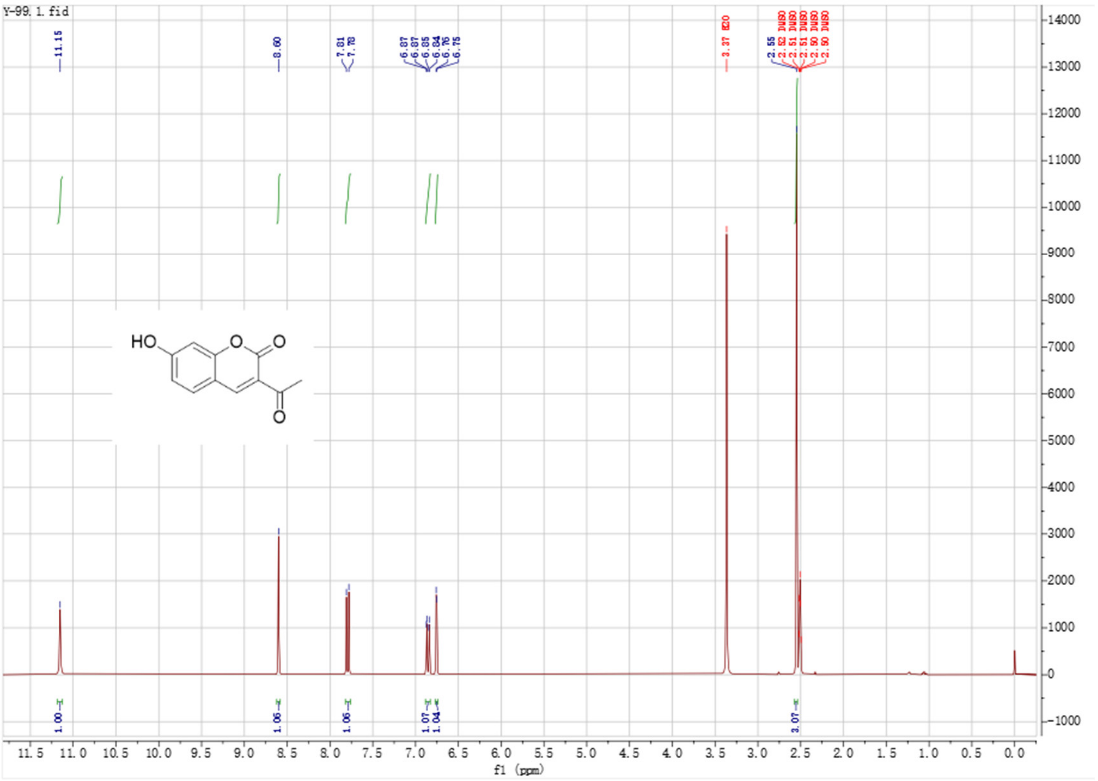

Figure S1. ^{1}H NMR spectrum of Compound 2.

Synthesis of 3-Acetyl-7-hydroxy-2H-chromen-2-one (Compound 2) 2, 4-Dihydroxybenzaldehyde (279 mg, 2 mmol) and Ethyl acetoacetate (253 µl, 2 mmol) were dissolved in ethanol (6 ml), followed by a few drops of piperidine as a catalyst, and the reaction mixture was returned to 78°C for 2 h to cool. Pour cold, dilute hydrochloric acid, filter the precipitate, rinse the precipitate with water, and recrystallize the purified residue from methanol to get the product. The product is a light yellow crystal (286 mg, 70%).^{1}H NMR (300 MHz, DMSO-d_6) δ(ppm): 11.15 (s, 1H), 8.60 (s, 1H), 7.78-7.81 (d, J = 8.4 Hz, 1H), 6.84-6.87 (dd, J_1 = 8.7 Hz, J_2 = 2.4 Hz, 1H), 6.75-6.76 (d, J = 2.1 Hz, 1H), 2.55(s, 3H). HRMS $C_{11}H_8O_4$, m/z: $[M+H]^+$ calcd 205.05, found 205.05.

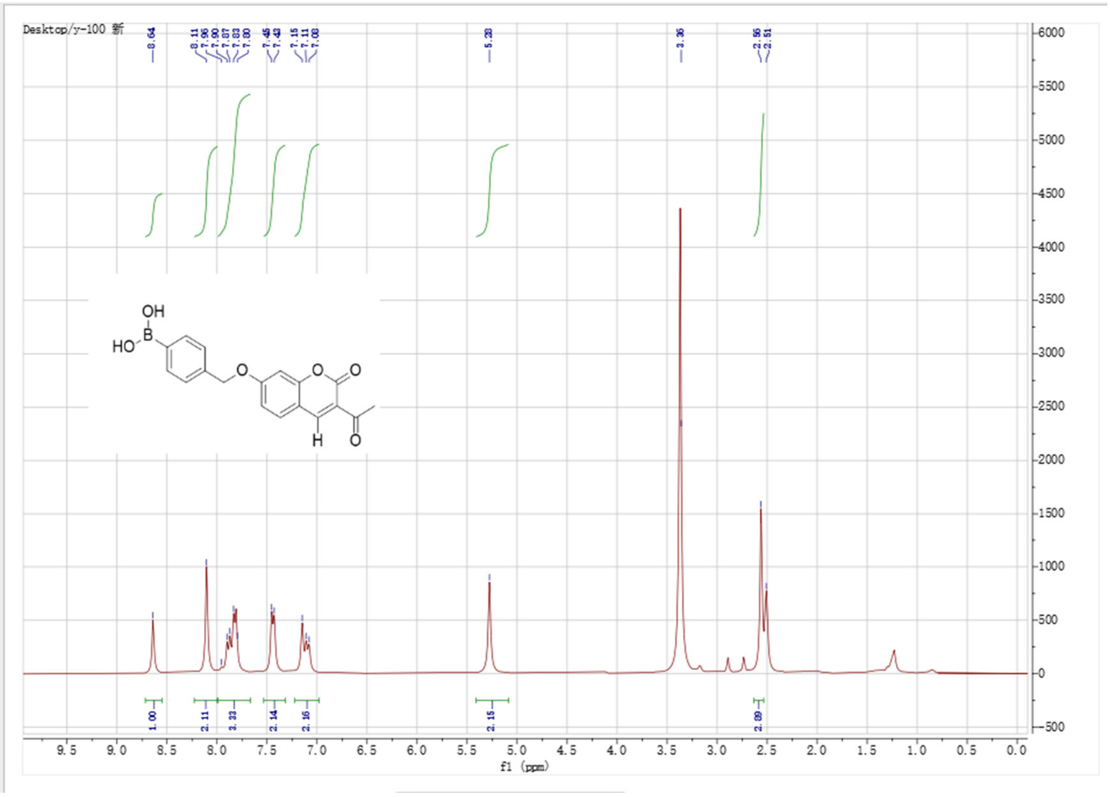

Figure S2. ^{1}H NMR spectrum of YXSH.

Synthesis of 3-Acetyl-7- [(4-boronyl) method] -2H-1-benzopyran-2-one (compound YXSH), 3-Acetyl-7-hydroxy-2H-chromen-2-one (202 mg, 1 mmol), 4-(Bromomethyl)phenylboronic acid(219 mg, 1 mmol), Anhydrous K_2CO_3(963 mg, 7 mmol) and acetone (15 ml) were added to the flask, the reaction mixture was reflow at 55℃ for 14 h, the reaction mixture was cooled and filtered, the solvent was removed by spin evaporation, DCM extraction was carried out, the organic phase was cleaned in saturated salt water and then dried on anhydrous sodium sulfate, and then filtered, and the volatiles were removed under vacuum. The residue was purified by silica gel column chromatography to obtain a crude product, which was then recrystallized with DCM n-hexane to produce a bright yellow powder (179 mg, 53%).^{1}H NMR (300 MHz, DMSO-d_6) δ(ppm): 8.64 (s, 1H), 8.11 (s, 2H), 7.80-7.96 (m, 3H), 7.43-7.45 (d, J = 7.5 Hz, 2H), 7.11 (t, J = 10.8 Hz, 2H), 5.28 (s, 2H), 2.56 (s, 3H). HRMS $C_{18}H_{15}BO_6$, m/z: $[M]^+$ calcd 338.10, found 338.34.

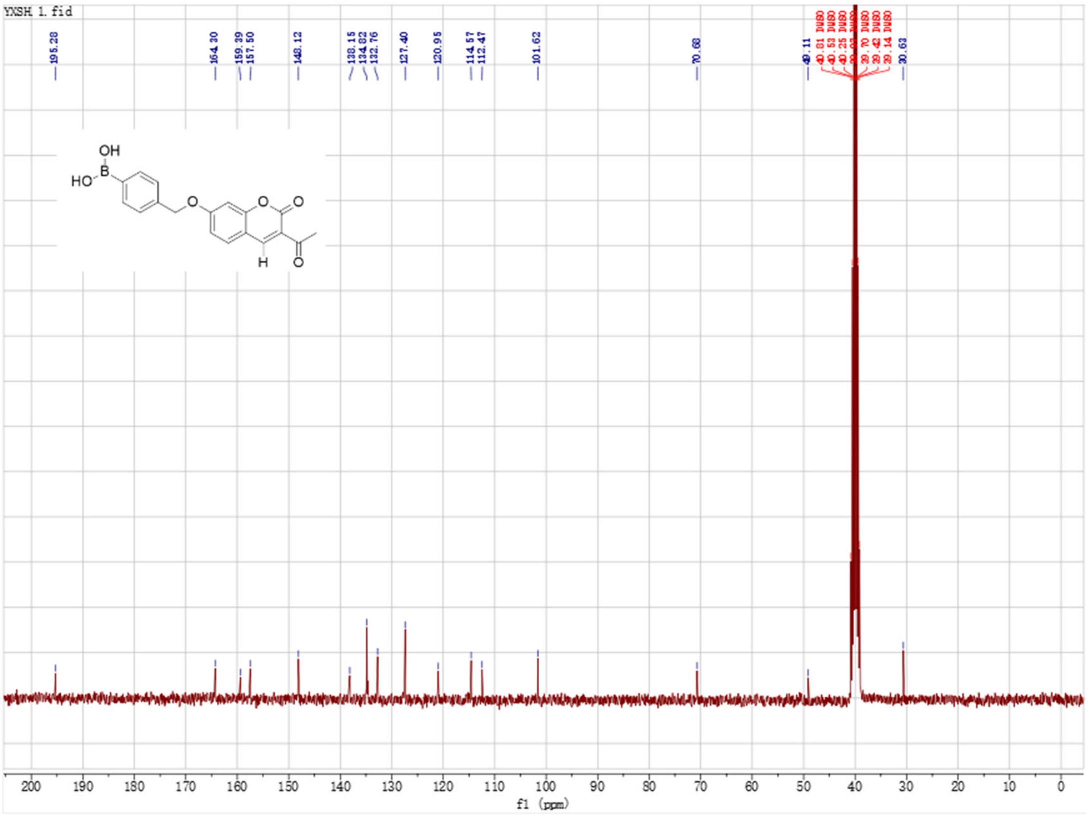

Figure S3. ^{13}C NMR spectrum of YXSH.

^{13}C NMR (300 MHz, DMSO-*d*$_6$) δ(ppm):195.28, 164.30, 159.39, 157.50, 148.12, 138.15, 134.82, 132.76, 127.40, 120.95, 114.57, 112.47, 101.62, 70.68, 49.11, and 30.63.

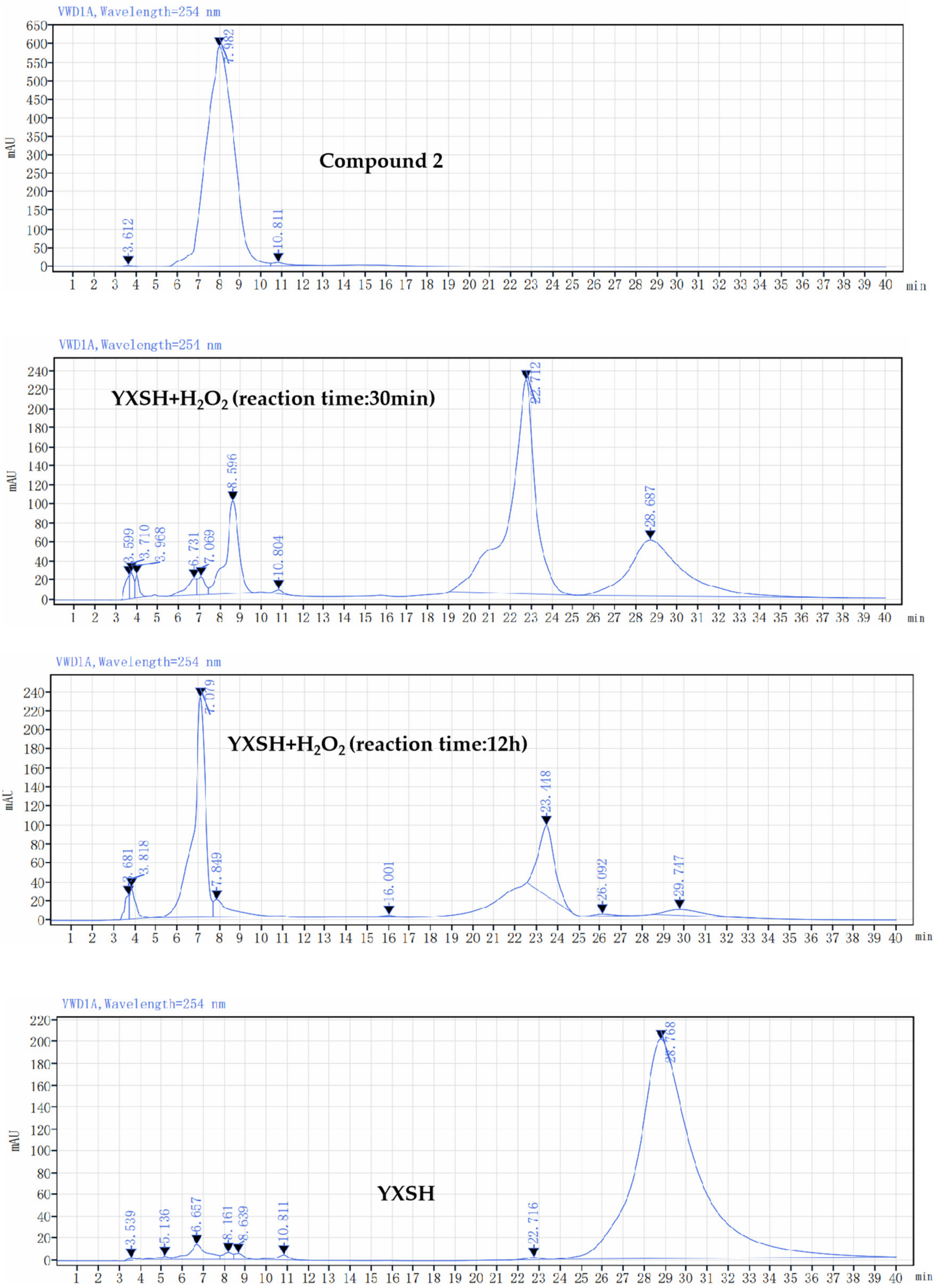

Figure S4. Liquid chromatography of YXSH, YXSH treated with H_2O_2, and Compound 2.
